# An implantable system for long-term assessment of atrial fibrillation substrate in unanesthetized rats exposed to underlying pathological conditions

**DOI:** 10.1038/s41598-020-57528-3

**Published:** 2020-01-17

**Authors:** Hadar Klapper-Goldstein, Michael Murninkas, Roni Gillis, Wesam Mulla, Eran Levanon, Sigal Elyagon, Ronen Schuster, Dor Danan, Hagit Cohen, Yoram Etzion

**Affiliations:** 1 Cardiac Arrhythmia Research Laboratory, Department of Physiology and Cell Biology, Faculty of Health Sciences, Ben-Gurion University of the Negev, Beer-Sheva, Israel; 2 Regenerative Medicine & Stem Cell Research Center, Ben-Gurion University of the Negev, Beer-Sheva, Israel; 3 Department of Clinical Biochemistry & Pharmacology, Faculty of Health Sciences, Ben-Gurion University of the Negev, Beer-Sheva, Israel; 4 Beer-Sheva Mental Health Center, Ministry of Health, Anxiety and Stress Research Unit, Faculty of Health Sciences, Ben-Gurion University of the Negev, Beer-Sheva, Israel

**Keywords:** Atrial fibrillation, Atrial fibrillation

## Abstract

Atrial fibrillation (AF) is a progressive arrhythmia with underlying mechanisms that are not fully elucidated, partially due to lack of reliable and affordable animal models. Here, we introduce a system for long-term assessment of AF susceptibility (substrate) in ambulatory rats implanted with miniature electrodes on the atrium. Rats were subjected to excessive aldosterone (Aldo) or solvent only (Sham). An additional group was exposed to myocardial infarction (MI). AF substrate was tested two- and four-weeks post implantation and was also compared with implanted rats early post-implantation (Base). Aldo and MI increased the AF substrate and atrial fibrosis. In the MI group only, AF duration was correlated with the level of atrial fibrosis and was inversely correlated with systolic function. Unexpectedly, Shams also developed progressive AF substrate relative to Base individuals. Further studies indicated that serum inflammatory markers (IL-6, TNF-alpha) were not elevated in the shams. In addition, we excluded anxiety\depression due to social-isolation as an AF promoting factor. Finally, enhanced biocompatibility of the atrial electrode did not inhibit the gradual development of AF substrate over a testing period of up to 8 weeks. Overall, we successfully validated the first system for long-term AF substrate testing in ambulatory rats.

## Introduction

Atrial fibrillation (AF) is a growing epidemic, entailing substantial economic costs, morbidity and mortality^1,2^. The pathophysiology of AF is multi-factorial in nature. It involves sources of sustained rapid electrical activity that can trigger arrhythmic episodes as well as pathological mechanisms which alter the electrical and structural substrate for AF in the atrial tissue^3–7^. A full understanding of the molecular mechanisms by which various underlying conditions and factors converge to progressively promote AF substrate is still lacking^8–10^. Drugs aimed to target the atrial remodeling (upstream therapies) are attractive new options to prevent AF perpetuation. However, early pre-clinical testing of such drugs is currently difficult due to the absence of reliable and affordable animal models.

Traditionally, AF-related models have relied solely on large animals exposed to atrial tachypacing or heart failure^11^. However, over the last two decades, rodents have been increasingly used to study various mechanistic aspects in the pathophysiology of AF^12–18^, and the possibility of using rodents to test new therapies seems attractive. However, several technical limitations constrain the widespread utility of rodents in AF research. Particularly, the small and delicate rodent atria render the implantation of chronic pacing and recording electrodes challenging. As a result, AF substrate is evaluated using either *ex-vivo* preparations or invasive studies in deeply anesthetized rodents that are sacrificed at the end of the procedure. Indeed, repeated testing of AF substrate development over time utilizing measurements under physiological conditions, (i.e. in the unanesthetized state) has not been reported thus far to the best of our knowledge. Recently, Hulsmans *et al*.^19^ reported the development of a miniaturized pacemaker for long-term pacing studies in rodents. However, although this technically-advanced system may open remarkable new opportunities, the currently introduced pacer could not be used for the electrophysiological (EP) testing, which was done conventionally under deep anesthesia as a single terminal procedure.

Our laboratory has developed the Miniature-Bipolar Hook Electrode (MBHE), which is adapted specifically for cardiac pacing and EP of rodents. This tool enabled efficient EP studies in anesthetized rodents^20–22^ as well as important, previously technically-challenging EP studies in unanesthetized rats and mice^20,23,24^. Specifically, MBHE is a powerful tool to assess the supraventricular EP of rodents consistently and precisely^20,21^. Moreover, by using a bi-atrial pacing and recording device, our laboratory previously performed atrial EP measurements in unanesthetized rats and was able to characterize in details the effect of short-term atrial tachypacing (up to 24 h) on atrial electrical remodeling^20^. Recently, we also managed to examine the effect of atrial tachypacing maintained for up to seven consecutive days, on the developed AF susceptibility (AF substrate) of rats and mice^25^.

In the present study, based on the MBHE system for unanesthetized rodents^23–25^, we aimed to leverage our implantable device for long-term testing of AF substrate development in fully ambulatory rats over a period of four weeks. As a proof of concept, we exposed the rats to two important underlying pathological factors known to promote AF substrate formation, namely hyperaldosteronism and ischemic cardiomyopathy^26,27^. Our findings clearly demonstrate the utility of the system for repeated AF substrate testing. In addition, the obtained experimental findings expose some important properties of the system itself as well as the underlying pathological factors that were selected for testing.

## Methods

### Animals

The study was carried out in strict accordance with the Guide for the Care and Use of Laboratory Animals of the National Institute of Health. All animals studies reported in this study were approved by the institutional ethics committee of Ben-Gurion University of the Negev, Israel. Adult male Sprague-Dawley rats (250–350 g) were obtained from Envigo Laboratories (Jerusalem, Israel). The animals were kept under standardized conditions throughout the study, according to home office guidelines: 12:12 light:dark cycles at 20–24 °C and 30–70% relative humidity. Animals were free-fed autoclaved rodent chow and had free access to reverse osmosis filtered water. The animals were monitored on a daily basis for signs of stress or inappropriate weight loss, according to guidance from the Ben-Gurion University veterinary services (assured by the Office of Laboratory Animal Welfare, USA (OLAW) #A5060-01, and fully accredited by the Association for Assessment and Accreditation of Laboratory Animal Care International (AAALAC)). At the end of all experiments animals were euthanized under deep anesthesia.

**Figure 1:**
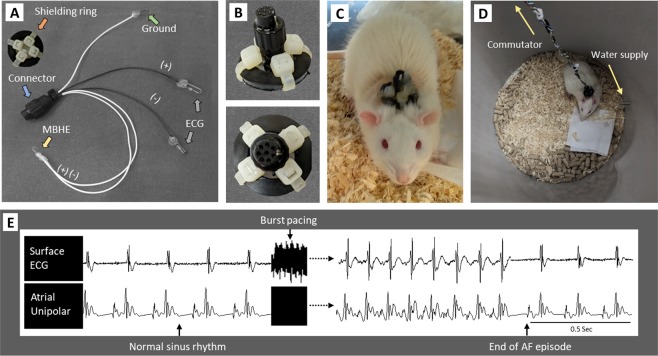
Implanted device and experimental apparatus for long-term AF substrate analysis in rats. (**A**) Photograph of an implanted device. The MBHE is implanted on the RA for atrial pacing (yellow arrow). Peripheral ECG and ground poles are implanted subcutaneously (grey & green arrows). Signal from one pole of the MBHE recorded against an ECG pole (Atrial-unipolar) is used to acquire high resolution readings of the atrial activity. A shielding ring (orange arrow) with four plastic restraints is used to prevent extraction of the device over time. (**B**) Side view and upper view of the shielding ring over the back connector. After implantation, the ring is inserted over the connector, sutured to the skin and glued to the connector over four plastic restraints. (**C**) An implanted rat ambulatory in the cage between the electrophysiological studies. (**D**) An implanted rat connected to the pacing & recording apparatus for AF substrate evaluation. Recording amplifiers and pacers are connected to the rat through commutator and flexible electrical cable (arrow). The rat has free access to food and water. (**E**) Recording of an AF episode triggered by burst pacing. *Left*: Baseline ECG and atrial-unipolar recordings. *Middle*: a standard AF triggering burst (20Sec, 100 Hz, double threshold). *Right*: Post-burst traces of an AF episode and its conversion to sinus rhythm.

Overall the results are based on 77 rats successfully implanted with the atrial pacing & recording device. Failure of the initial procedure occurred in less than 10% of cases and included either periprocedural mortality or failure in the implantation of the atrial MBHE leading to absence of effective atrial pacing. During preliminary experiments we encountered failures to reach the final EP testing at 4 weeks in several occasions as a result of self-extraction of the back connector. This problem was totally resolved by the placement of a shielding ring over the back connector (Fig. 1). Initial experiments included 43 rats divided into 3 groups: Base group (n = 12) implant with the atrial pacing & recording device EP tested at 1 W only. These animals were thereafter used for atrial tachypacing experiments, which were recently reported^25^ and are beyond the scope of the current study. Sham group (n = 15) co-implanted with atrial pacing & recording devices and osmotic mini-pumps delivering solvent only (PEG-400). Aldosterone (Aldo) group (n = 16) co-implanted with atrial pacing & recording devices and osmotic mini-pumps delivering Aldosterone dissolved in PEG-400. An additional MI group (n = 14) included rats subjected to device implantation and left coronary artery ligation. An additional sham-operated group maintained two in a cage separated by a mesh barrier, was also included (n = 7) in order to test the contribution of social isolation as a source of intrinsic AF substrate. Finally, a new MBHE composed of medical-grade silicone and Platinum-Iridium (Pt-Ir) poles was also evaluated (n = 13). Apart from the base group, all of the reported EP results are only for rats that could be tested serially at 2 W and 4 W. For the new MBHE, reported results include serial tests up to 8 W from the initial surgery.

### The implantable device and EP apparatus

The implantable EP device for rats and the implantation technique, were previously described in detail by our group^23,24^. Briefly, the implantable device is composed of an 8-pin ‘female’ connector that is attached by highly flexible insulated electrical wires (AS155-36, Cooner wires, Chatsworth, CA) to a single miniature-bipolar hook electrode (MBHE). The MBHE contains a distal head with two sharp tungsten pins (100 μM in diameter, A-M Systems, Sequim, WA) that are curved and isolated by an insulated coating (transparent melting glue) up to their tips (Fig. 1A). By means of small lateral thoracotomy the MBHE can be fixed on the selected epicardial surface without the need for additional suturing^20,22–24^. Three additional single lead electrodes are utilized for grounding and peripheral ECG measurements (Fig. 1A). Before implantation, the 8-pin ‘female’ connector and the distal part of the MBHE were covered with latex to prevent direct contact with the skin and subcutaneous tissue during the procedure. Electron beam radiation was applied for sterilization of the device before its use. For device implantation animals were anesthetized (IM ketamine/xylazine 75/5 mg kg^−1^) and mechanically ventilated. Under sterile conditions and constant heating, the MBHE was implanted on the RA, the peripheral electrodes were positioned in the animal’s back and the 8-pin ‘female’ connector was exteriorized through the skin. A shielding ring with four plastic restraints was used to prevent extraction of the device over time (Fig. 1A,B). After implantation, the ring was inserted over the connector, sutured to the skin and glued to the connector over four plastic restraints. This procedure largely prevented cases of connector extraction during the long periods in which the rats were fully ambulatory (Fig. 1C). Post-operative recovery and analgesia were performed as described previously^24^.

For each AF substrate evaluation animals were placed in dedicated recording chambers where the 8-pin connectors were attached to the pacing and recording apparatus through elastic electrical cables. The proximal part of the elastic cable was connected to a multi-channel commutator (PLA-SL12C/SB, PLASTICS One Inc., CA) enabling free rotational movements of the rat in the cage without effects on the electrical connections. In each animal the two poles of the atrial MBHE were electrically connected to an optically isolated pacing unit (STG4002-16mA, Multichannel systems, Reutlingen, Germany) and two of the peripheral electrodes were utilized for ECG recordings (Amplifier 1700, A-M systems, Carlsborg, WA). In addition, differential recording between one pole of the MBHE and one of the ECG leads was used to obtain an ECG signal in which the atrial activity could be seen with high resolution (Atrial unipolar, Fig. 1D). Signals were filtered (1 Hz–1 KHz) and sampled to the PC at a digital sample rate of 2 KHz using an A/D converter (PCI-6024E, National Instruments, Austin, TX). A LabView-based program (National Instruments) controlled data acquisition and electrical stimulation.

### Design of the new Platinum-Iridium based MBHE

In the last section of this paper, experiments with a new type of MBHE are described. This MBHE is composed of Platinum-Iridium (Pt-Ir) electrical poles, miniature stainless steel hooking pins and medical-grade silicon. In order to fabricate this MBHE, the distal end of two flexible electrical wires coming from the implantable device (AS155-36, Cooner wires, Chatsworth, CA) were inserted in their distal end with Pt-Ir rods (2 mm in length, 200 μM in diameter). The Pt-Ir rods were then placed parallel to each other on the bottom of a cubic shaped Teflon template (5 mm length × 2.5 mm width × 2 mm height), which was subsequently filled with medical-grade silicon (MED-6219P, Nusil, CA). Following incubation of the silicon for 24 hours at room temperature, the electrode was extracted from the Teflon template and two stainless steel insect pins (26002-10, Fine Science Tools, Vancouver, Canada) were angled and embedded into the silicon to form a double hook. An additional shorter pin was inserted in the rear part of the electrode to prevent extraction of the MBHE from the cardiac tissue over time. Following preparation electrodes were excessively washed with 70% ethanol and were sterilized with electron beam radiation before use.

### AF substrate evaluation

Each AF substrate evaluation was done following overnight adaptation of the animal to the EP cage according to standard burst pacing protocol. The protocol was performed during the early daytime hours of the circadian cycle when animals were inactive. Each AF substrate evaluation protocol included 20 consecutive atrial pacing bursts (20 S, double diastolic threshold, 10 ms cycle length-CL). Analysis of AF inducibility was performed by calculating the percentage of positive episodes (defined as episodes lasing more than 1 S). Analysis of AF duration was performed by calculating “*Total AF duration score*” as detailed below. Arrhythmic episodes were identified by recordings demonstrating more than one atrial signal per QRS and atrial signal which was clearly different in morphology in comparison with the regular atrial signal during sinus rhythm. We did not discriminate between regular and irregular atrial rhythms in the current analysis. Arrhythmic episodes lasting more than 5 minutes were aborted using short (1 S) pacing bursts of increasing intensity until sinus rhythm was restored. The minimal time between pacing bursts was 1 minute. If an episode of more than 1 minute was detected, the delay from the end of this episode to the next pacing burst was equal to the duration of the episode.

#### Total AF duration score

Although most of the arrhythmic episodes induced by burst pacing in rats are rather short and convert spontaneously to sinus rhythm, the analysis of total AF duration is sometimes complicated by sporadic long lasting episodes that can last several minutes and more. While the latter episodes are not common, they can have a profound effect on the analysis. In order to be able to evaluate changes in AF duration that attenuate the contribution of these long sporadic episodes, the “total AF duration score” (range 0–5) was defined as follows: 0 – negative (less than 1 S); 1- episode duration between 1–5 sec; 2- episode duration between 5–20 sec; 3- episode duration between 20–60 sec; 4- episode duration between 60–240 sec; 5- episode duration of more than 240 sec. The sum of the scores of all episodes in each AF substrate analysis is calculated as the total AF duration score.

### Osmotic mini-pumps and coronary artery ligation

Osmotic mini-pumps (ALZET pump Model 2004) were implanted subcutaneously in the lower back following implantation of the EP device. Aldosterone (Sigma-Aldrich) was dissolved in polyethylene glycol 400 (PEG 400, Sigma-Aldrich) to maintain a release rate of 1.5 µg/h^26^. Sham operated animals were inserted with pumps containing PEG 400 only. In the MI group ligation of the LAD was performed after the device implantation, by left lateral thoracotomy. The pericardium was removed and the proximal left anterior descending coronary artery was permanently occluded with an intramural suture (6-0 polypropylene). MI was confirmed acutely by the presence of regional cyanosis in the myocardial surface and later on by the development of systolic dysfunction and scar formation.

### Echocardiography

Echocardiography measurements were performed with a dedicated system for rodents (Vevo 3100, FUJIFILM VisualSonics, Canada) as previously described by our group^28^. During the procedure the rats were lightly anesthetized by 1.5% isoflurane/O2 mixture and were placed in a left decubitus position on a heating pad to maintain a rectal temperature of ~37 °C. The duration of the whole echocardiography procedure was restricted to 15 minutes. 2D images of the left ventricle were taken in parasternal long and short axis views. Long and short axis M-mode images were obtained at the mid papillary muscles area with cursor penetration at the papillary muscle tip. LV end-diastolic diameter (LVDed) and LV systolic diameter (LVDes) were obtained from the long-axis M-mode trace. LV fractional shortening (FS - %) was calculated according to (LVDed−LVDes)/LVDed × 100. LV ejection fraction (LVEF) was calculated using planimetry as follows: EF = 100 × (LVD3ed − LVD3es/LVD3ed). Evaluation of EF within the MI group was performed using Simpson volume analysis (VEVO LAB, VisualSonics, Canada). For this purpose three 2D short axis images were obtained: proximal - just below the mitral valve, mid- at the papillary muscle level and apical- at the apex level. Simpson EF was calculated as: (SIMPS. Area distal + SIMPS. Area mid + SIMPS. Area prox). *Length/3. All measurements were averaged for three consecutive cardiac cycles and performed by an experienced technician who was blinded to the treatment groups.

### Serum and histology measurements

At the terminal procedure, blood samples were collected from the tail artery of each rat. Samples were centrifuged (14000 RMP, 4 °C) and serum was collected and stored at −80 °C.

Rat serum TNFα (Rat TNF-α ELISA MAX™ Deluxe, range: 15.6–1000 pg\mL) and IL-6 (LEGEND MAX™ Rat IL-6, range: 18.8–1,200 pg/mL) were measured according to manufacturer’s instructions (all from BioLegend, San Diego, CA). Aldosterone levels were determined by competitive chemiluminescent immunoassay (CLIA) technology using LIAISON® XL analyzer (Diasorin Inc., Saluggia, Italy) in the central endocrine laboratory of Soroka university medical center, Beer-Sheva, Israel.

Hearts were isolated for histological analysis. Left atrial (LA) and short axis slices of ventricles were excised, fixed in 4% paraformaldehyde for 24 hours, embedded in paraffin and sectioned into 5 µm thickness slices. Sections were deparaffinized in xylene, rehydrated in a descending alcohol sequence and transferred into distilled water. Masson trichrome (MT) staining was performed according to the manufacturer’s protocol (04-010802, Bio Optica, Milano, Italy). The stained sections were scanned with a panoramic scanner (panoramic MIDI II, 3DHISTH, Budapest, Hungary) and analyzed automatically by customized software (Quant center 2.0 software, 3DHISTH). Myocardial fibrosis was reported as the collagen volume fraction (CVF) and calculated with the equation of total collagen area/total field area (including both perivascular and interstitial collagen). The reader was blinded to group assignment. For the MI rats infarct size (%) was calculated as follows: Rats were sacrificed and hearts were cut into four short axis sections. Photos were taken from the sections and transformed to ImageJ for analysis. Infarct size (%) was calculated as the ratio between the circumference of the infarct zones and the total circumference of the LV in the four sections.

### Statistical analysis

Values are expressed as mean ± SE. For histological and echocardiographic measures, data were compared by one-way ANOVA with Dunnett multiple comparison post-test. AF substrate parameters did not have normal distribution and were therefore analyzed using nonparametric testing: Comparisons between 2 W and 4 W within each group was done using Wilcoxon matched-pairs signed rank test. Comparisons between similar weeks in two treatment groups was done using Mann-Whitney test. Comparisons of all the data to the 1 W data of the baseline group was done using Mann-Whitney test with Bonferroni correction for multiple comparisons. For the new MBHE, repeated measure comparisons between 1 W, 2 W, 4 W and 8 W were performed by Friedman test with Dunn’s multiple comparison post-test. The criterion for significance was set at p < 0.05. Unless otherwise stated p-values are displayed graphically as follows: *p < 0.05, **p < 0.01, ***p < 0.001, ns = not significant. For 0.06 ≥ p > 0.05 the p-values are indicated in the figures but results are regarded as non-significant. In all of the figures the number of analyzed rats (n) is shown for each condition.

## Results

### Repeated AF substrate analysis in unanesthetized rats

**Figure 2:**
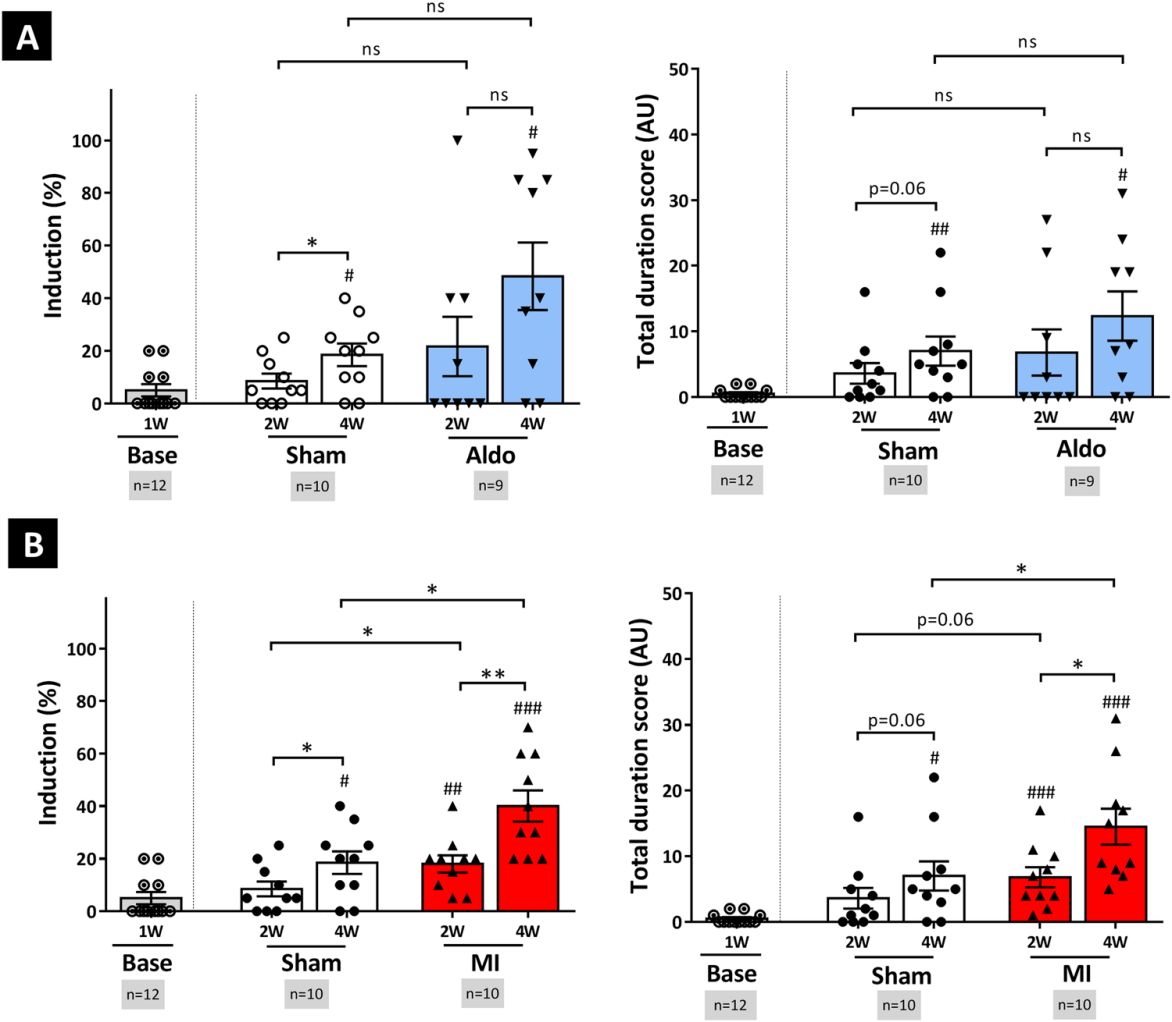
Serial measurements of AF substrate in the unanesthetized state. (**A**) AF substrate analysis in rats implanted with the pacing and recording device: Base- rats implanted with the device and tested in the system following 1 W recovery period only. Sham- rats subjected to device implantation and insertion of osmotic pumps releasing solvent only. Aldo- rats subjected to device implantation and insertion of osmotic pumps releasing aldosterone (1.5 mcg/hour). In the last two groups, AF substrate was tested 2 and 4 weeks post implantation. *Left:* AF inducibility *Right:* Total AF duration score (see methods section for details). Note a very low AF substrate in the Base group, but prominent and progressive AF substrate in the Aldo group as well as in the sham-operated group. Comparison between Sham and Aldo at same time frame (2 W or 4 W) was performed using Mann-Whitney test. Comparison between 2 W and 4 W within each group was performed with Wilcoxon test. #, ##, ### - Comparison to the Base group with Bonferroni’s correction for multiple comparisons. ns = not significant. (**B**) Similar representation as in A comparing the Base and Sham group to an MI group subjected to device implantation and left anterior descending artery (LAD) ligation.

As a first step in the characterization of our new system, we evaluated the AF substrate of healthy rats implanted with the MBHE system and subjected to 1 W recovery period from the initial surgery (Fig. 2A,B, Base group). All twelve rats that were used for this preliminary evaluation demonstrated very low AF inducibility and AF duration score. These findings suggest that the implantation surgery per-se does not promote AF substrate formation. As already detailed in the methods, the Base group was later on used for atrial tachypacing studies which were recently published^25^.

In the next stage, rats chronically exposed to excessive aldosterone levels (Aldo), an established trigger of AF substrate in rats^26,29^, were followed for four weeks in comparison with sham-operated animals. Interestingly, in both groups AF substrate progressively developed over time leading to significantly increased induction and total AF duration score at four weeks in comparison with the Base animals. However, the variability of the AF substrate parameters in the Aldo group was remarkable, with some animals demonstrating high AF substrate and others demonstrating very low AF substrate parameters under similar conditions (Fig. 2A,B). Of note, a pilot analysis at the end of four weeks confirmed the presence of increased serum aldosterone levels in Aldo treated animals relative to Shams (see Supplementary Figure S1). To further substantiate our findings regarding AF substrate formation with the MBHE system we subjected an additional group of rats to MI, another clinically relevant trigger known to promote the AF substrate of rats^30^. Indeed, MI led to progressive increase of the AF substrate parameters over time and in contrast to Aldo, it also led to increase of the AF substrate parameters in comparison with the Sham group (Fig. 2C,D).

**Figure 3:**
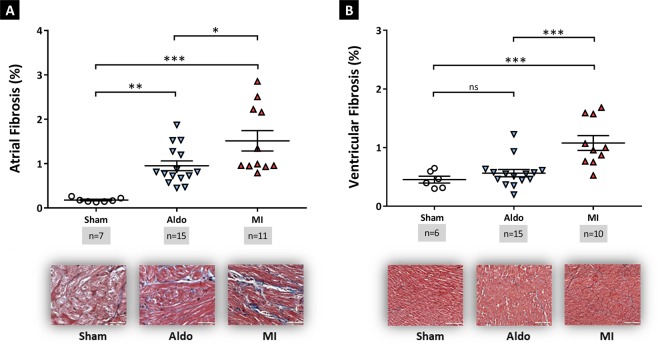
Structural remodeling in rats exposed to aldosterone and myocardial infarction. (**A**) Lt. atrial fibrosis evaluated using Masson’s trichrome (MT) staining. *Upper*: summarizing dot plot of quantitative image processing analysis. *Lower*: representative photomicrographs for each condition. Statistical analysis was performed with 1-way ANOVA. Note increased left atrial fibrosis in Aldo model and even more so in the MI model relative to Sham. (**B**) Analysis of ventricular fibrosis (Similar representation as in A). Data represent combined measurements of right and left ventricular zones. In the MI rats only non-infarcted zones were analyzed. As expected, interstitial fibrosis was increased in the MI group.

**Figure 4:**
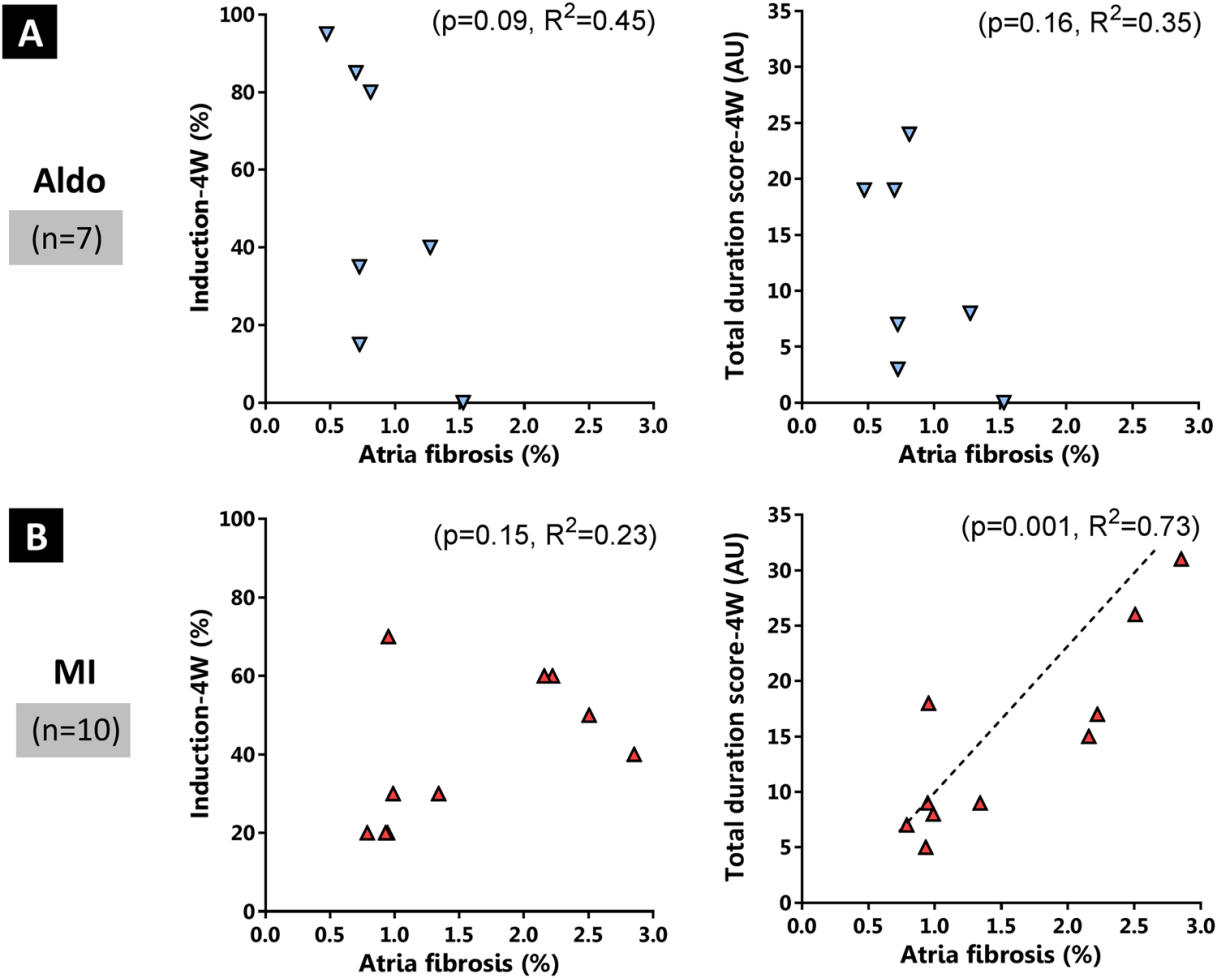
Analysis of the interconnection between structural remodeling and AF substrate. (**A**) Scatter plots correlating AF substrate parameters at 4 W (*Left*: Induction, *Right*: Total duration score) with LA fibrosis (MT staining) in the Aldo group. Linear regression results are shown for each plot. Note for both parameters the absence of significant correlation with the level of atrial fibrosis. (**B**) Similar representation as in A for the MI group. Note a clear correlation of the total duration score with the level of fibrosis in this group.

**Figure 5:**
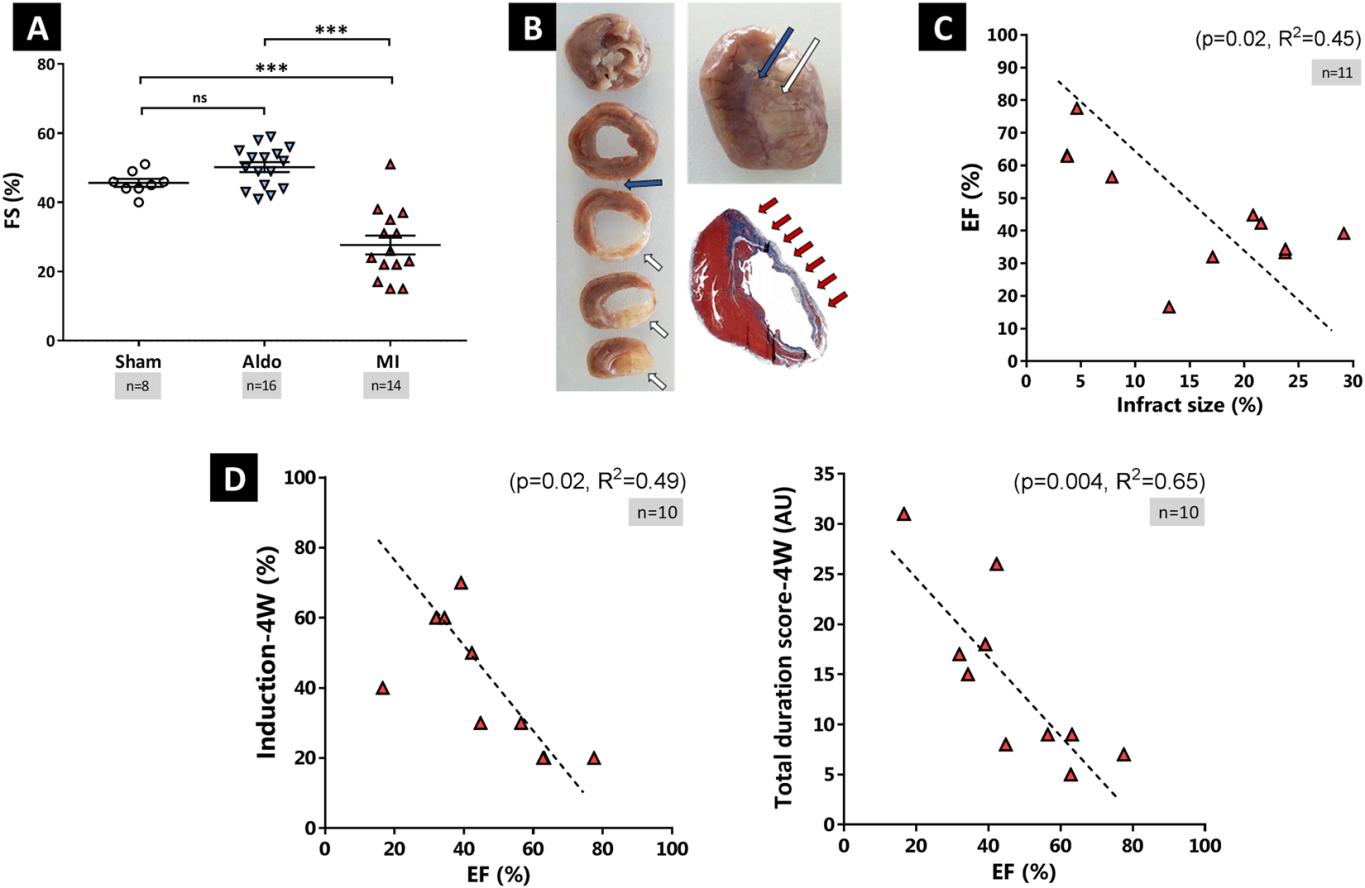
Analysis of the interconnection between LV systolic function and AF substrate. (**A**) Comparison of Fractional Shortening (FS %) between the Sham, Aldo and MI groups. Note the expected decrease of FS in the MI group. (**B**) *Upper right:* Frontal view of an infarcted heart. Blue arrow indicate the ligation suture. White arrow mark the infarcted zone. *Lower right:* Example of a section stained for collagen content using Masson’s trichrome. Infarcted area is marked by red arrows. *Left:* Short axis sections. The ligation suture is marked by blue arrow, the infarcted zones are marked by white arrows. Infarct size was calculated as described in the methods. (**C**) Scatter plot correlating Infarct size with left ventricular ejection fraction (EF %). Note significant inverse correlation. (**D**) Scatter plots correlating AF substrate parameters at 4 W (*Left*: Induction, *Right*: Total duration score) with EF. Linear regression results are shown for each plot. Note clear inverse correlation of both parameters.

In order to examine the relations between the above EP findings and the atrial structural remodeling of the rats, LA fibrosis was quantified following MT staining. As expected, atrial fibrosis was markedly increased in the Aldo group and even more so in the MI group relative to the Sham animals (Fig. 3A). Ventricular fibrosis was elevated in the MI group only. Surprisingly, our analysis revealed no association between the level of LA fibrosis and the AF substrate parameters in the Aldo group (Fig. 4A). In contrast, clear association between LA fibrosis and the total AF duration score was found in the MI group (Fig. 4B). Additional analysis of the MI group indicated that the systolic dysfunction of these rats (Fig. 5A and Supplementary Table 1S) clearly correlated with both MI size and AF substrate parameters (Fig. 5). Overall, our findings indicate that although structural remodeling is an overt finding in both Aldo and MI rats, it seems to indicate increased AF substrate only in the MI group in relation with MI size and impairment of LV systolic function (see discussion).

### Characterization of intrinsic AF substrate in the implanted rats

In contrast to the very low AF substrate of the Base group following 1 W recovery period, an overt finding of the noted above experiments was the progressive development of AF substrate in the sham group over time (Fig. 2). This phenomenon appears to occur spontaneously, without exposing the implanted rats to any extrinsic stimulus mimicking a pathology. However, the contribution of possible unintentionally applied insult had to be excluded.

First, TNF-a and IL-6 were tested in the serum to rule out systemic inflammation. Both inflammatory cytokines were found to be below the detected levels (15.6 pg/mL and 18.8 pg/mL, respectively) in all tested animals in the sham group (n = 14). In addition, as already described (Fig. 3), histological analysis did not reveal increased LA fibrosis in the sham group, as would be expected in rats exposed to systemic inflammation^31,32^.

**Figure 6:**
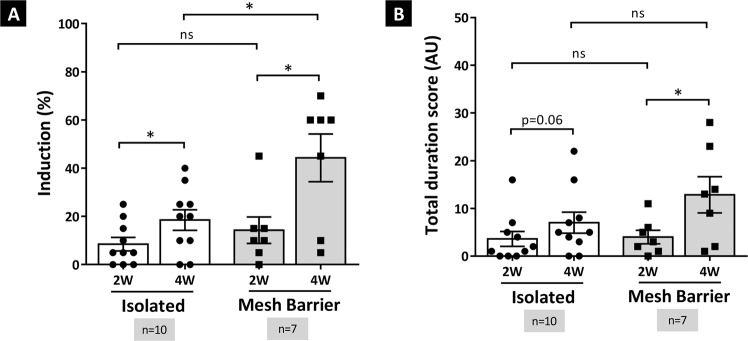
Effect of chronic social isolation on AF substrate. Comparison of AF substrate between the original sham-operated group (similar data as in Fig. 3), maintained in separate cages (Isolated) and an additional group of sham rats in which chronic social isolation was prevented by placing two rats in each cage separated by a mesh barrier preventing the rats from damaging each others’ connectors (see details in text). (**A**) AF inducibility. (**B**) Total AF duration score. Note that in contrast to our prediction, prevention of social isolation did not prevent AF substrate formation. On the contrary, the mesh barrier significantly increased induction at 4 W.

Next, we evaluated the possible role of chronic social isolation in the observed AF substrate formation. Our MBHE system normally requires holding of the rats in separate cages in order to prevent damage to the back connector by the cage mates. Since chronic social isolation is a known trigger of anxiety\depression in rats^33^, we raised the possibility that this factor might be involved in the observed AF substrate formation. To test this option in a direct manner we used a simple manipulation allowing socially rewarding interaction between rats through a mesh-barrier^34^. First, we directly confirmed that behavioral parameters of anxiety\depression are markedly inhibited in rats interacting through a mesh-barrier (Supplemental text and Supplementary Figure S2). Based on these findings, an additional group of sham-operated rats were maintained for 4 W in cages containing a mesh-barrier. Interestingly, EP analysis revealed that this manipulation could not reduce the AF substrate (Fig. 6). On the contrary, at 4 W animals in the non-isolated group had small but significant increase in AF induction compared to the original (Isolated) sham group.

### AF substrate with the new Platinum-Iridium based MBHE

**Figure 7:**
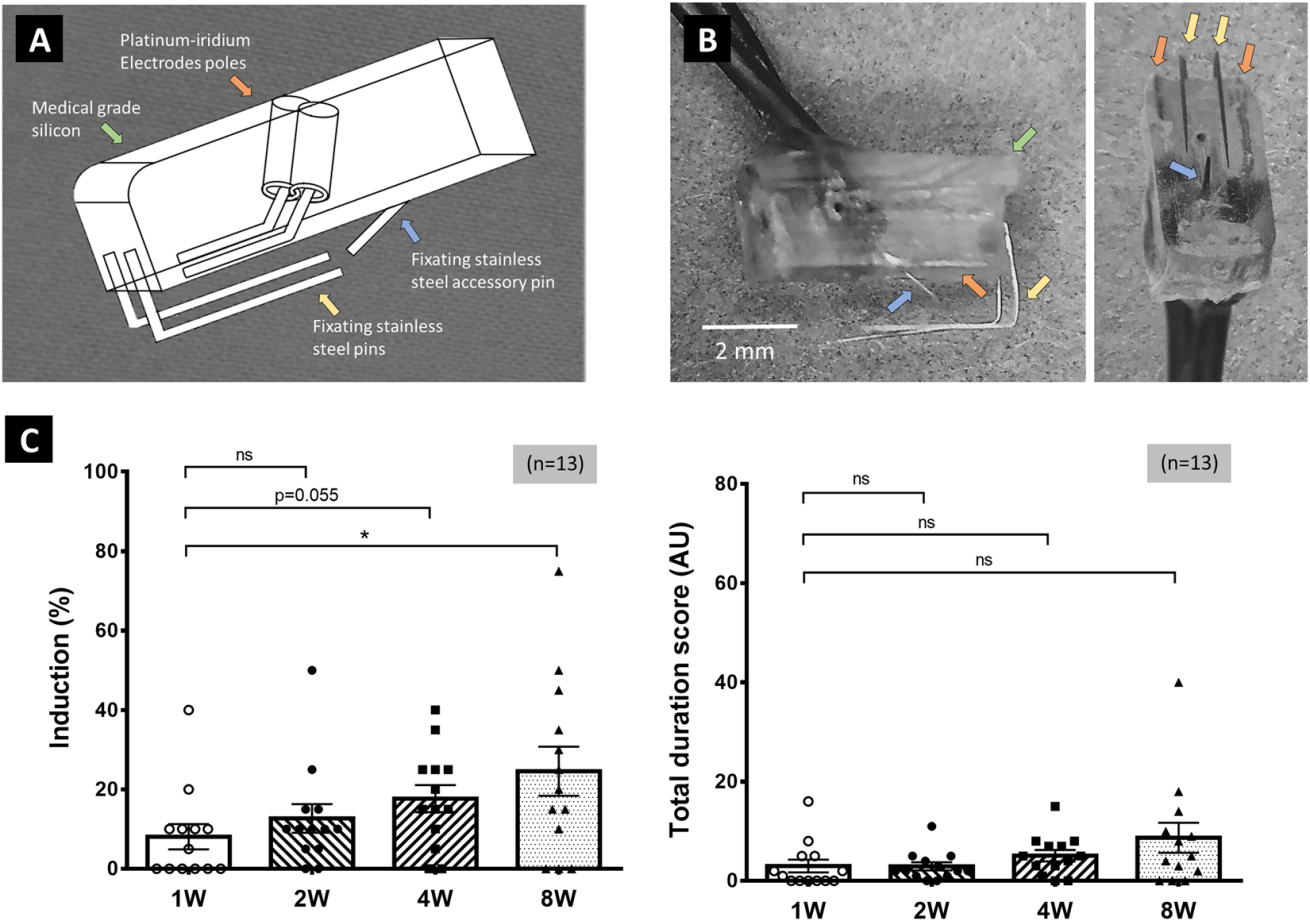
Effect of the new platinum-iridium based MBHE on the AF substrate. (**A**) Schematic presentation and (**B**) photographs of the new MBHE made of medical grade silicone (green arrow) embedded with electrical poles made out of platinum-iridium (orange arrows). The delicate fixating pins (yellow arrows) and rear accessory pin (lite blue arrow) inserted into the tissue, are made of stainless steel instead of tungsten which was recently reported to have unfavorable properties as a chronic implant^35^. (**C**) AF substrate parameters over time in rats implanted with the new MBHE presented in A and B. AF substrate was evaluated 1 W, 2 W, 4 W and 8 W following implantation. *Left:* AF inducibility *Right:* Total AF duration score. Comparison between the different time frames was done using Friedman test with Dunn’s multiple comparison post-test. Note that the new MBHE could not abolish the progressive increase of AF inducibility over time. However, the duration score did not change significantly over this long period of testing. For comparison with the original MBHE see Supplemental Figure 1S.

Based on the above results, we next hypothesized that intrinsic AF substrate of the sham-operated rats might be related to local interaction between components of the MBHE and the RA tissue, possibly triggering local toxic\inflammatory effects over time. Specifically, we suspected that tungsten, which was recently suggested to have unfavorable long-term effects as a chronically implanted material^35^, was involved in this phenomenon. To address this possibility, a new type of MBHE was fabricated. In this type of MBHE, tungsten was replaced by Pt-Ir electrode poles and miniature stainless steel pins were used for hooking of the MBHE on the atria (Fig. 7A,B). In addition, as detailed in the methods section, medical grade silicon was used in this electrode instead of the transparent melting glue, which was used in the original MBHE. Thus, all of the components of the new electrode were made out of inert biocompatible components.

To comprehensively address spontaneous AF substrate formation with the new MBHE, repeated EP studies were performed 1 W, 2 W, 4 W and 8 W from the initial implantation. Indeed, the new electrodes were highly efficient in terms of pacing and 100% of implanted rats (n = 13) could be followed for the whole 8 W period without a significant change in capture threshold. However, progressive AF substrate formation was still present with new MBHE electrode (Fig. 7C) and direct comparison of the results in the first 4 W showed no significant benefit of the new device compared to the original tungsten-based MBHE (Supplementary Figure S3).

## Discussion

The present study successfully introduced an implantable device and EP apparatus for repetitive AF substrate evaluation in unanesthetized freely moving rats. We demonstrated the stability of our system over a period of several weeks and the ability to combine the system with clinically relevant pathological factors that have been shown to promote AF substrate formation in humans as well as in rats. The experimental findings in regard to Aldo and MI offer interesting insights in regard to the association between atrial structural remodeling and AF substrate formation under these underlying conditions. In addition, the progressive AF substrate development that we characterized in Sham animals is a major finding that should have important implications for any future rodent studies with atrial implantable systems. These issues will be discussed in details bellow.

In several previous studies we have already demonstrated the utility of the implantable MBHE system in gaining new insights on the electrophysiology of unanesthetized freely moving rodents^20,23–25^. However, this study is the first in which the system was successfully employed for repeated EP evaluations over a period of several weeks (up to 8 W with the new MBHE, Fig. 7). A rather simple technical improvement that enabled this achievement was the use of a shielding ring that was sutured to the skin around the back connector (Fig. 1A,B). This addition efficiently prevented device extraction over time by the rats. Apart from this modification, our previously developed MBHE system was well-suited for the long-term EP studies of the present work. Repeated AF substrate evaluations were efficiently obtained from the RA electrode while leaving the LA tissue free of surgical operation for later histological analysis. Atrial-unipolar recordings which we utilized to view the atrial activity of the rats were far superior to the peripheral recordings in terms of atrial signal detection and analysis (Fig. 1E) as we also reported recently (See Fig. 1C in Mulla *et al*.^25^). However, a clear limitation of the current setup was the inability to obtain additional EP parameters such as atrial refractoriness (AERP). This is related to the fact that only two electrical poles are present in the current MBHE design and such setup does not allow clear detection of the atrial signal during pacing^20^. Clearly, a future improvement of the system enabling the insertion of two pairs of electrical poles (one pair for pacing and additional one for recording) would be of great value. Such an improvement might also enable measurements of conduction velocity, another critical parameter which could not be addressed in our unanesthetized rats using the current setup.

Although it is hard for us to give precise estimation of the full costs per implantable device (including the worktime, which is not uniform), we can clearly state that the cost of the raw materials for a single device is in the order of few tens of dollars and the procedures for fabrication are quite easily acquired by most students\technicians with good manual skills. Thus, we believe (and hope) that this tool would be affordable for the great majority of academic laboratories in the EP field and will be utilized extensively for mechanistic and proof of concept studies, before getting to highly demanding large animal experiments.

The selection of Aldo and MI was based on the strong evidence for the involvement of these etiological factors in the development AF in humans and animal models^26,30^. The MI group mimics a heart failure model of AF substrate formation. Thus, the observed correlations between the AF substrate parameters and atrial fibrosis or LV systolic dysfunction in this group are to be expected^36^. In contrast, the large variance in the developed AF substrate of the Aldo group as well as the absence of correlation between the AF substrate and the level of LA fibrosis are surprising. High Aldo states have been associated with increased prevalence of AF and experimental results also indicate increased density of its receptors in the fibrillating atria^37–40^. Aldo promotes atrial fibrosis and blockade of its receptors can efficiently block this effect^37,41–44^. Interestingly, while excessive Aldo promotes AF substrate locally in the atria, its systemic effects can promote insulin resistance, resistant hypertension, tissue generation of oxygen free radicals and systemic inflammation^45^. Thus, the involvement of Aldo in the pathogenesis of AF might comprise complex interactions between local and systemic effects.

In rats Reil *et al*.^26^, demonstrated that 8 W of exposure to Aldo causes atrial fibrosis, myocyte hypertrophy, and conduction disturbances. However, their *ex-vivo* EP studies did not identify AERP changes following exposure to Aldo and the authors concluded that fibrosis leading to decreased conduction velocity is the main factor promoting AF substrate. In contrast Lammers *et al*.^29^, did identify action potential duration shortening following 4 W of exposure to Aldo, which was associated with increased expression of Kir2.1 and Kv1.5 potassium channels in isolated atria of the Aldo-treated rats. Our current findings strongly suggest that although atrial fibrosis is indeed a uniform finding in Aldo-treated rats, factors other than fibrosis probably contribute to the development of AF substrate. Nevertheless, our current study was not specifically designed to mechanistically address this issue and further insights on this important issue will have to await future analysis.

The most surprising finding of the current work was the gradual development of intrinsic AF substrate in our sham-operated rats over time. This intriguing finding led us to several additional experiments in order to try to reveal possible source\s for this phenomenon. The results strongly argue against the involvement of systemic inflammation as an underlying mechanism based on direct serum measurements of inflammatory cytokines as well as the absence of atrial fibrosis which is expected to occur in the presence of systemic inflammation^31,32,46^. Post-operative AF due to the thoracotomy we performed in the implantation surgery is another option that should be considered. However, post-operative AF typically demonstrate peak incidence between days 2 and 4 after surgery and frequent recurrences, especially during the first postoperative week^47^. This time course in in clear contrast with the very low AF burden that we consistently encountered 1 W post-op, which gradually developed and increased over time. Thus, although we cannot totally exclude this option, the kinetics of AF development in the Sham animals makes this possibility quite unlikely.

Depression has been lately shown to be associated with an increased clinical risk of atrial fibrillation, although a causal relation has not been elucidated so far^48^. Anxiety\depression related to chronic social isolation is a well know phenomenon in rodents including rats^33,49^. Thus, we invested substantial effort in delineating the possible role of social isolation as a cause for the intrinsic AF substrate. Using a simple manipulation allowing socially rewarding interaction between rats through a mesh barrier^34^, we confirmed a substantial reduction in anxiety\depression. However, we found that this inhibition of anxiety\depression slightly augmented rather than reduced the developed AF substrate. The possible mechanism\s of this augmentation can only be speculated and require further and more detailed characterization in future studies. Nonetheless, our data can unequivocally rule out the possibility that chronic social isolation may have a causative roll in the intrinsic AF substrate development of rats implanted with our MBHE system.

Finally, in order to examine the possibility of local interaction between the implanted electrode and the RA, we designed, fabricated and comprehensively tested a new type of MBHE with components that are not expected to promote toxic effects or increased local inflammation. While, the new electrode was highly effective in EP terms, it did not prevent the progressive development of AF substrate over time. It is possible that the local injury induced by the delicate stainless steel pins of the new MBHE is enough to cause some non-uniform conduction leading to AF substrate formation. However, the gradual development of the substrate over a period of many weeks is somewhat inconsistent with this option. Another possibility is that the local load of the implanted electrode on the contracting RA caused stretch related remodeling over time^50^. Sterile pericarditis due to local removal of the RA pericardium is another option that should be considered. In any case, on a practical level it appears that the intrinsic AF substrate that we characterized here is unavoidable under the current conditions and should be taken into consideration in any future study utilizing this or similar system for mechanistic or pharmacological studies of the AF substrate in rats.

## Supplementary information

Supplementary Information
