# Supplementary material for: An implantable system for long-term assessment of atrial fibrillation substrate in unanesthetized rats exposed to underlying pathological conditions
Source: Sci Rep. 2020 Jan 17;10:553. doi: 10.1038/s41598-020-57528-3 (PMC6969190; doi:10.1038/s41598-020-57528-3)
Supplement: Supplementary file 1 — Supplementary Information [file 41598_2020_57528_MOESM1_ESM.pdf]

SUPPLEMENTAL MATERIAL

An implantable system for long-term assessment of atrial fibrillation substrate in unanesthetized rats exposed to underlying pathological conditions

Hadar Klapper-Goldstein[1,2*], Michael Murninkas [1,2*], Roni Gillis [1,2], Wesam Mulla[1,2] Eran Levanon[1,2], Sigal Elyagon [1,2], Ronen Schuster [3], Dor Danan[4], Hagit Cohen[4] and Yoram Etzion [1, 2]

Affiliations:

[1] Cardiac Arrhythmia Research Laboratory, Department of Physiology and Cell Biology, Faculty of Health Sciences, Ben-Gurion University of the Negev, Beer-Sheva, Israel.

[2] Regenerative Medicine & Stem Cell Research Center, Ben-Gurion University of the Negev, Beer-Sheva, Israel.

[3] Department of Clinical Biochemistry & Pharmacology, Faculty of Health Sciences Ben-Gurion University of the Negev, Beer-Sheva, Israel.

[4] Beer-Sheva Mental Health Center, Ministry of Health, Anxiety and Stress Research Unit, Faculty of Health Sciences, Ben-Gurion University of the Negev, Israel.

Supplemental methods for mesh barrier experiments:

The experiments testing the behavioral effects of the mesh barrier procedure were part of a larger independent study that included a jugular vein catheter implantation surgery for purposes that are beyond the scope of this paper. All procedures were carried out under strict compliance with ethical principles and guidelines of the NIH Guide for the Care and Use of Laboratory Animals. All treatment and testing procedures were approved by the Animal Care Committee of Ben-Gurion University of the Negev, Israel (IL-09-06-2014).

Animals:

A sample of 23 adult male Sprague-Dawley rats (Envigo Laboratories, Jerusalem, Israel) weighing 190-220 gm were habituated to housing conditions for at least seven days. During this habituation period, three rats were housed per cage. After the catheter implantation surgery rats were housed either in isolation or in social groups (3 rats/cage) for 14 days prior to the start of the behavioral testing (Supplemental Figure S2-A). Throughout the experiments, the rats were maintained in a temperature controlled room (21±0.5 ºC) on a reverse 12 light–dark cycle, a 12:12 light-dark cycle (lights off at 07:00 p.m.; luminous emittance during the light phase: 200G50 lx), with unlimited access to food and water. All procedures were performed during the resting phase of the rats, between 07:30 and 18:30. Rats were handled daily commencing the week prior to experimental procedures.

Experimental procedures:

Following surgery, rats were distributed into three different housing conditions:

1. Control group (CONT): group housed in the cages (n = 8). 2. Isolation + standard condition group (SC): rats were housed singly in standard condition cages (Supplemental Figure S2-B, n = 7). 3. Isolation + mesh barrier group (MB): rats were housed singly in opaque plastic bins with restricted access to another rat through a mesh barrier (Supplemental Figure S2-C, n = 8). The standard condition cages consist of a 45 X 28 C 20 cm high, polysulfone box (Tecniplast). The

mesh barrier cages consist of a 40 X 28 X 20 cm high transparent Perspex box. Each chamber contained a removable partition that separated the chamber into two equal-sized compartments. The mesh barrier was made of wire grid mesh (1.5 × 1.5 cm), allowing the rats to insert their noses into the adjacent compartment. The group-housed cages were 56 cm×38 cm×20 cm high. After 14 days of a post-surgical recovery period, the behavioural assessments were conducted, first in the elevated plus maze (EPM) paradigm and 1 h later in the acoustic startle reaction (ASR) paradigm.

For the jugular vein catheter surgery rats were anaesthetized with a combination of ketamine (70 mg/kg) and xylazine (6 mg/kg) i.p. The right jugular vein was exposed and a polyurethane cannula (inner diameter 0.63 mm, outer diameter 1.02 mm, Instech, Plymouth Meeting, PA) was inserted into the vessel until it lay close to the entrance of the right atrium. The cannula was prefilled with pyrogen-free heparinized (10 IU/ml) isotonic saline. The free end of the cannula was exteriorized through an incision between the shoulderblades and then capped. Jugular cannulae were manually flushed daily by withdrawing 0.2 ml of blood and replacing the volume with 0.3 ml of sterile heparinized saline (0.2 ml to replace blood volume; 0.1 ml to clear blood from cannulae) to maintain patency of the cannulae.

Behavioural measurements:

The behavior of rats was assessed in the EPM and ASR paradigms, as described previously and as briefly detailed below (Cohen *et al*, 2013 [1]).

Elevated plus-maze (EPM): The maze was a plus-shaped platform with two opposing open arms and two opposing closed arms (closed arms surrounded by 14 cm high opaque walls on three sides) (File, 1993 [2]). Rats were placed on the central platform, facing an open arm, and were allowed to freely explore the maze for 5 minutes. Each test was videotaped and the behavior of the rat was subsequently scored by an independent observer. Arm entry was defined as entering an arm with all four paws. At the end of the 5 min test period, the rat was removed

from the maze, the floor was wiped with a damp cloth, and any faecal boluses removed. An anxiety index, which integrates the elevated plus-maze (EPM) behavioral measures, was calculated for each rat according to the following formula:

$$Anxiety\ \ Index = 1 - \left[\frac{\left(\frac{time\ \ spent\ \ in\ \ the\ \ open\ \ arms}{total\ \ time\ \ in\ \ the\ \ maze} \right) + \left(\frac{number\ \ of\ \ entries\ \ to\ \ the\ \ open\ \ arms}{total\ \ exploration\ \ of\ \ the\ \ maze} \right)}{2} \right]$$

Anxiety Index values thus range from 0 to 1, with higher values reflecting a more anxiety-like behavior. The use of anxiety index has been validated in prior studies (e.g. (Cohen *et al*, 2013 [1])).

Acoustic startle response (ASR): Startle responses were measured by using two ventilated startle chambers (SR-LAB system, San Diego Instruments, San Diego, CA). The SR-LAB calibration unit was used routinely to ensure consistent stabilimeter sensitivity between test chambers and over time. Each Plexiglas cylinder rested on a platform inside a soundproof, ventilated chamber. Movement inside the cylinder was detected by a piezoelectric accelerometer below the frame. Sound levels within each test chamber were measured routinely with a sound level meter (Radio Shack) to ensure consistent presentation. Each test session began with a 5-min acclimatization period to background white noise of 68 dB, followed by 30 acoustic startle trial stimuli presented in 6 blocks (110 dB white noise of 40 ms duration and 30 or 45 sec inter-trial interval). Two behavioral parameters were assessed: (a) the mean startle amplitude (averaged over all 30 trials); and (b) the percent of startle habituation to repeated presentation of the acoustic pulse. For the latter, the percent change was calculated between the response to the first and last (6th) blocks of sound stimuli, as follows:

$$Percent\ \ Habituation = 100 \times \left[\frac{(average\ \ startle\ \ amplitude\ \ in\ \ Block\ \ 1) - (average\ \ startle\ \ amplitude\ \ in\ \ Block\ \ 6)}{(average\ \ startle\ \ amplitude\ \ in\ \ Block\ \ 1)} \right]$$

Supplemental results for mesh barrier experiments:

In the elevated plus maze test, there were significant differences between the groups in terms of the time spent in the open arms ($F(2,20)=7.6$, $p<0.0035$) (Supplemental Figure S2-D), the time

spent in the closed arms ($F(2,20)=7.4$, $p<0.004$) (Supplemental Figure S2-E), the number of entries to the open arms ($F(2,20)=10.2$, $p<0.0009$) (Fig. 1F) and the anxiety index (Fig.1H). Rats that had been isolated under standard conditions exhibited significantly decreased overall time spent in the open arms of the maze (Post-hoc Bonferroni test: $p<0.006$), in the number of entries to the open arms ($p<0.0015$) and in the anxiety index ($p<0.001$) as compared to control group. Rats that had been isolated in mesh barrier cages significantly corrected isolation-induced anxiety-like behavior, resulting in a significant relative increase in total time spent in the open arms of the maze ($p<0.015$) in number of entries to the open arms ($p<0.006$) and in anxiety index ($p<0.0015$). In effect, there were no significant differences between the control group and the mesh barrier group. No differences were observed in total exploration of the maze between groups, suggesting overall anxiety-like behavior and avoidance of exploration in the open arms, as opposed to a nonspecific impairment of locomotion.

Supplemental Figure S2-I shows the mean startle amplitude of rats in response to the 30 startle-pulses. There were significant differences between the groups ($F(2,20)=28.8$, $p<0.0001$). Post hoc Bonferroni test revealed that isolation in both standard cages and in mesh barrier cages significantly increased the mean startle amplitude as compared to controls ($p<0.0001$ and $p<0.009$, respectively). Moreover, rats that had been isolated in mesh barrier cages significantly attenuated the hyperarousal response, eliciting a relative decrease in mean startle amplitude compared to the mesh barrier group ($p<0.001$).

Supplemental Figure S2-J shows the “percent habituation”-parameter of the startle response in all groups. Percent habituation differed significantly among the groups ($F(2,20)=6.7$, $p<0.006$). Post hoc Bonferroni test revealed that isolation under standard conditions caused a significant deficit in the habituation of the acoustic startle response compared with control rats ($p<0.008$). Isolated in mesh barrier cages reversed this habituation deficit to a significant degree ($p<0.03$). There were no significant differences between the control group and the mesh barrier group. To

summarize, isolation in mesh barrier cages corrected anxiety-like behaviors on the EPM and startle habituation, and had a partial effect on amplitude of ASR, relative to control group.

Supplemental references for mesh barrier experiments:

1. Cohen H, Matar MA, Joseph Z (2013). Animal models of post-traumatic stress disorder. *Curr Protoc Neurosci* **Chapter 9**: Unit9 45.

2. File SE (1993). The interplay of learning and anxiety in the elevated plus-maze. *Behavioural brain research* **58**(1-2): 199-202.

Supplemental Figures and Tables:

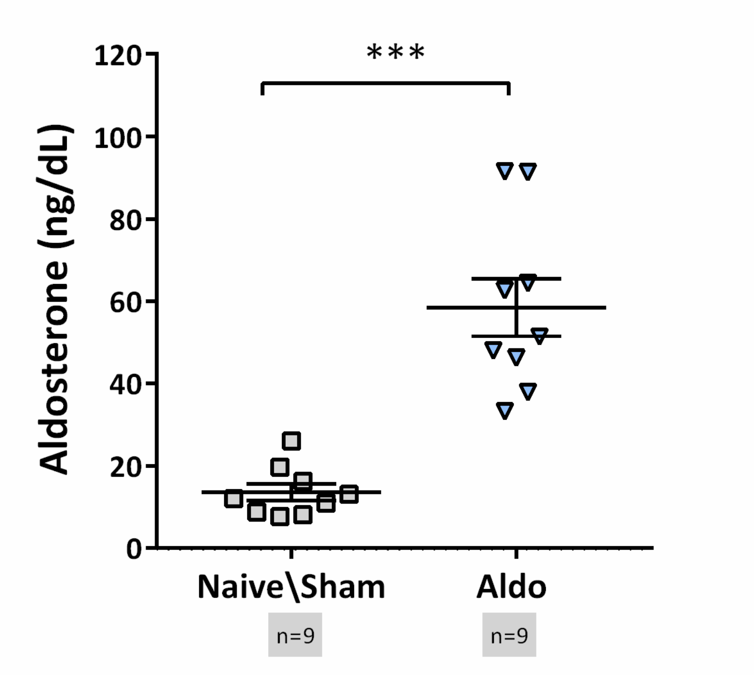

Supplemental Figure S1: Serum aldosterone levels in Naïve and Sham animals (Naïve\Sham) vs. Aldo treated animals (Aldo). Serum samples were obtained during the terminal procedure, four weeks post implantation and demonstrate effective induction of hyperaldosteronism in the Aldo treated group. Naïve (n=5) and Sham (n=4) animals did not differ significantly in their Aldo levels and were thus combined together. Statistical comparison was performed using conventional unpaired student's t-test.

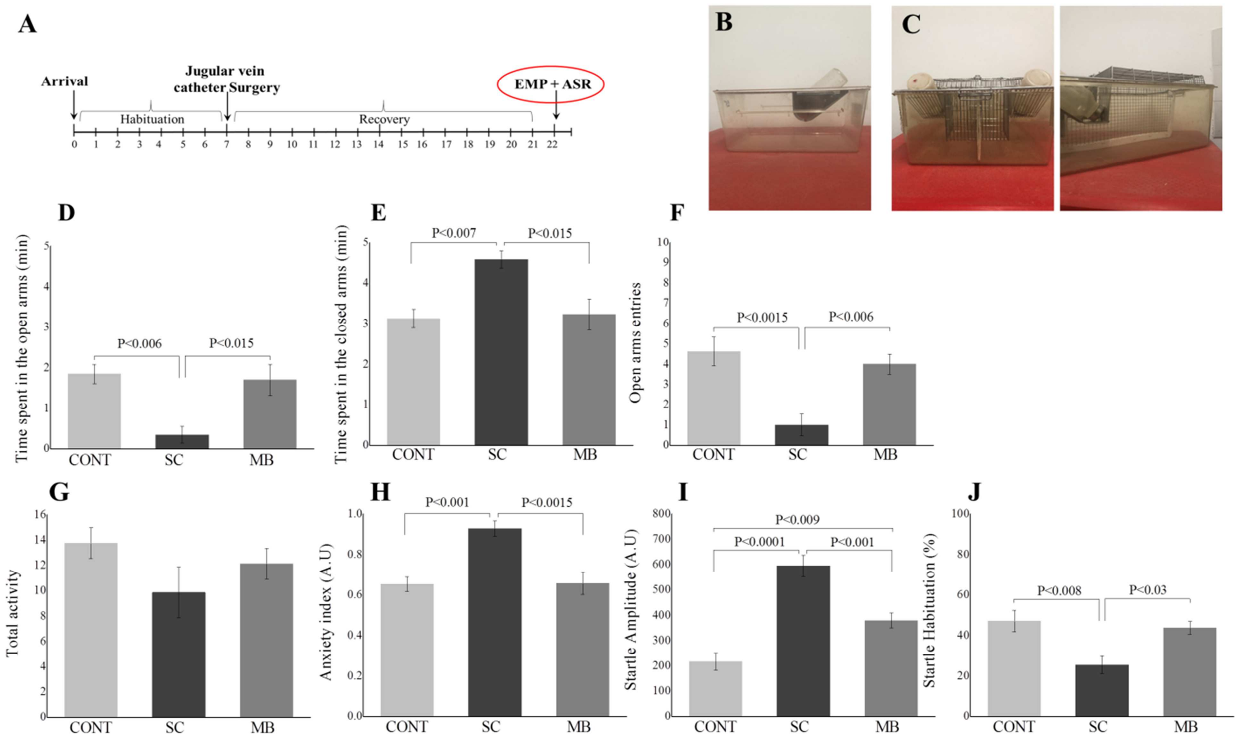

Supplemental Figure S2: Mesh barrier cages reduce anxiety-like behavior. **A**. The experimental protocol is depicted in the top panel. **B.** Standard cage **C**. A special cage with the mesh barrier. **D**. Time spent in the open arms of the maze, **E**. Time spent in the closed arms of the maze, **F**. the number of entries to the open arms, **G**. Total activity on the maze, **H**. Anxiety index, **I**. Startle amplitude and **J**. Startle habituation. Overall, rats that had been isolated in mesh barrier cages showed significantly reduced anxiety-like behavior in the elevated plus maze and in the acoustic startle response paradigms. Bars represent mean ± S.E.M.

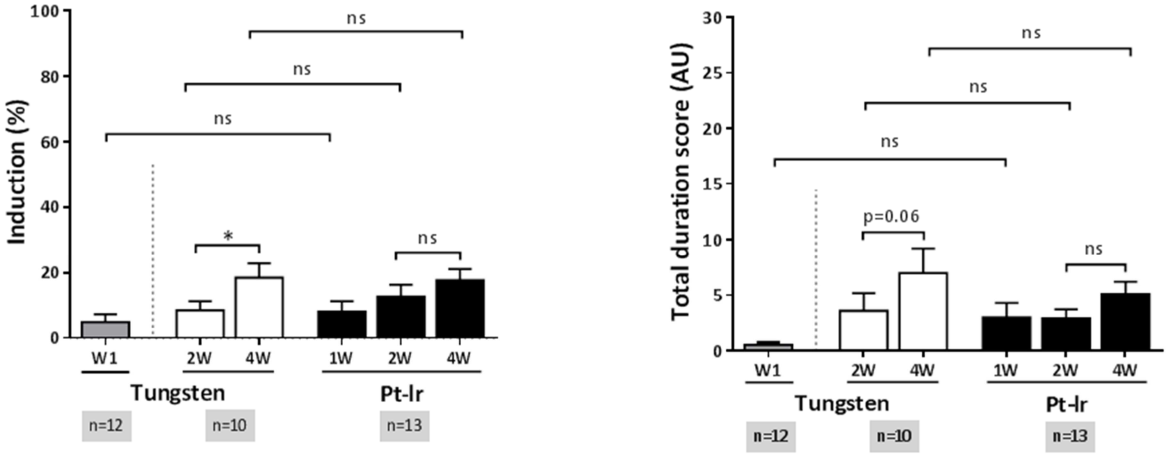

Supplemental Figure S3: Comparison between the original and the new type of the MBHE in regard to AF substrate formation. For the original MBHE (Tungsten) data is similar to the base and Sham groups in Fig. 3. For the new MBHE (Pt-Ir) AF substrate was evaluated 1W, 2W and 4W following implantation. *Left:* AF inducibility *Right:* Total AF duration score. Comparison between Tungsten and Pt-Ir at same time frames was performed using Mann-Whitney test. Note that the new MBHE could not abolish the development of AF substrate in sham operated animals.

Table S1 – Echocardiographic parameters

	**Sham (S)** n=8	**Aldo (A)** n=16	**MI (M)** n=14	**p**
Heart rate (b.p.m)	362.5±8.62	332.8±6.4 *	335±7.04	P=0.03
LVIDd (mm)	8.01±0.18	8.26±0.09	9.67±0.24 **#,$**	P<0.0001
LVIDs (mm)	4.39±0.12	4.13±0.13	7.02±0.39 **#,$**	P<0.0001
EF%	75±1.24	78.74±1.23	36.64±3.79 **#,$**	P<0.0001
FS%	45.63±1.18	49.63±2.23	27.64±2.72 **#,$**	P<0.0001

LVIDd = Left ventricular internal diameter - Diastolic; **LVIDs** = Left ventricular internal diameter-Systolic; **EF** = Ejection fraction; **FS** = Fractional shortening. p - Represent the one way ANOVA p-value. Post hoc comparison: ***** significance of Aldo vs. Sham, **#** significance of MI vs. Sham, **$** significance of MI vs. Aldo
